# Astragalus Polysaccharides Attenuate Postburn Sepsis via Inhibiting Negative Immunoregulation of CD4^+^CD25^high^ T Cells

**DOI:** 10.1371/journal.pone.0019811

**Published:** 2011-06-15

**Authors:** Qing-yang Liu, Yong-ming Yao, Yan Yu, Ning Dong, Zhi-yong Sheng

**Affiliations:** Department of Microbiology and Immunology, Burns Institute, First Hospital Affiliated to the Chinese PLA General Hospital, Beijing, People's Republic of China; Mayo Clinic, United States of America

## Abstract

**Backgroud:**

Astragalus polysaccharides (APS) isolated from one of the Chinese herbs, *Astragalus mongholicus*, are known to have a variety of immunomodulatory activities. However, it is not yet clear whether APS can exert an effect on the immune functions of regulatory T cells (Tregs). This study was carried out to investigate the effect of APS on the immune function of peripheral blood Tregs in postburn sepsis.

**Methodology/Principal Findings:**

BalB/c mice were randomly divided into six groups as follows: sham burn group, burn control(burn without infection animals) group, burn plus *P. aeruginosa* group, burn plus *P. aeruginosa* with APS (50 mg/kg) treatment group, burn plus *P. aeruginosa* with APS (100 mg/kg) treatment group, and burn plus *P. aeruginosa* with APS (200 mg/kg) treatment group, and they were sacrificed on postburn day 1, 3, 5, and 7, respectively, with seven animals at each time point. Magnetic microbeads were used to isolate peripheral blood Tregs and CD4^+^ T cells. Phenotypes were analyzed by flow cytometry, and cytokine levels were determined with ELISA. In the burn plus *P. aeruginosa* group, forkhead/winged helix transcription factor p3 (Foxp3) expression on CD4^+^CD25^+^Tregs were strongly enhanced in comparison to the sham group, and the capacity of CD4^+^CD25^+^Tregs to produce interleukin (IL)-10 was markedly increased. Administration of APS to inhibit CD4^+^CD25^+^Tregs could significantly decrease expression of Foxp3 on CD4^+^CD25^+^Tregs, and IL-10 production in burned mice with *P. aeruginosa* infection. At the same time, proliferative activity and expression of IL-2 and IL-2Rα on CD4^+^ T cells were restored. In contrast, anti-Toll-like receptor 4 (TLR4) antibody could block the effect of APS on Tregs immune function.

**Conclusion:**

APS might suppress CD4^+^CD25^+^Treg activity, at least in part, via binding TLR4 on Tregs and trigger a shift of Th2 to Th1 with activation of CD4^+^ T cells in burned mice with *P. aeruginosa* infection.

## Introduction

Severe infection can result in the suppression of one or more functions of the host immune system. Multiple mechanisms have been proposed to explain infection-induced immunosuppression, including an imbalance in the cellular helper T cell (Th)1/Th2 or cytokine profile, induction of anergy, depletion of effector cells, and, most recently, the activation of regulatory T cells (Tregs) [1]. The role of both naturally occurring CD4^+^CD25^+^Tregs and interleukin (IL)-10-secreting Tregs in infection has been the subject of several excellent recent reviews [2], [3]. However, it seems that its response to trauma, burns, hemorrhagic shock, and microbial infection is associated with only a transient proinflammatory period, followed by a more prolonged period of immune suppression [4], [5]. Based on an advanced understanding of cellular immunity, it has been recognized that naturally occurring CD4^+^CD25^+^ Tregs, which is a functionally distinct and mature subgroup of T cells, control various immune responses, including autoimmune disease, maintenance of homeostasis, tumor immunity, transplantation tolerance and other inflammatory disorders [6], [7], [8], [9]. Thus, recently an increasing interest has arisen in regard to the investigation of the biology of Tregs as well as their potential mechanisms underlying immune dysfunction following acute insults, infection, and severe sepsis or septic shock [10], [11]. Studies have revealed that Tregs are characterized by their ability to suppress proliferation and function of conventional effector T cells by various means [12], [13]. Tregs activity is cell-contact dependent and is mediated by increased cytokines such as IL-10 [14]. Forkhead/winged helix transcription factor p3 (Foxp3) is considered to be a “master gene” in controlling the development and function of these cells. Many reports have demonstrated the role of Treg in chronic infectious diseases, such as infections due to virus, fungus, leishmania, schistosome, and various microbacteria. In addition, Tregs might also be involved in the pathogenesis of acute infectious processes [15], [16], [17]. Thus, it is important for providing the appropriate administration of immunomodulator to maintain host immunologic balance in the setting of sepsis.

The dried root of *Astragalus membranaceus* (Huangqi) has a long history of medicinal use in traditional Chinese medicine. It is now commonly used as an immunomodulating agent in mixed herbal decoctions to treat common cold, diarrhea, fatigue and anorexia [18], and it is also prescribed to patients with cardiac diseases [19]. In recent years, radix *Astragalus membranaceus* has also been used to ameliorate the side effects of cytotoxic anti-neoplastic drugs. The active pharmacological constituents of radix *Astragalus membranaceus* include various polysaccharides, saponins and flavonoids [20]. Among these, Astragalus polysaccharides (APS) have been most widely studied, mainly on their immunopotentiating properties like stimulation of murine B-cell proliferation and cytokine production [21]. Apart from these actions, clinical studies also showed that APS could counteract the side effects of chemotherapeutic drugs, such as a significant amelioration in the degree of myelosuppression in cancer patients [22]. Nevertheless, it remains unclear that whether APS have potential suppressive effect on the peripheral blood Tregs with subsequent activation of T cells.

In *in vivo* studies, administration of APS or Huangqi extract markedly increased normal murine Th1/Th2 cytokine ratio [23]. This immunopharmacological profile of APS might be related to its beneficial effect on the diseases with imbalance of Th1/Th2 cytokine ratio, including severe thermal injury. However, it is not clear whether APS can suppress the activation of Tregs, and subsequently, regulate T cells after burn injury. The present study was performed with the purposes to: (1) investigate the potential role of APS in regulating Tregs and the influence of APS on CD4^+^ T cell-mediated immunity after burns with *Pseudomonas aeruginosa (P. aeruginosa)* infection *in vivo*; and (2) elucidate the underlying mechanism of APS on the Tregs, which might regulate CD4^+^ T lymphocyte immune function via down-regulating Toll-like receptor (TLR) 4 expression on Tregs followed by shifting of Th2 to Th1 *ex vivo*.

## Materials and Methods

### Drugs

APS (Lot number 021001B) for clinical application is composed of α-1,4 (1,6) glucan, arabinose-galactose polysaccharides, rhamnose-galacturonic acid polysaccharides, and arabinose-galactose protein polysaccharide with molecular weights of 20000–60000, and an endotoxin content less than 0.1 EU/mg.

### Mice

Male BALB/c mice were purchased from Laboratory Animal Center of Chinese Academy of Medical Sciences, Beijing. They were 6–8 weeks old, weighing 20±1 g. All animals were housed in separate cages in a temperature-controlled room with 12 hour (h) light and 12 h darkness to acclimatize for at least 7 days before being used. All experimental manipulations were undertaken in accordance with the National Institute of Health Guide for the Care and Use of Laboratory Animals, with the approval of the Scientific Investigation Board of the Chinese PLA General Hospital (The Permit Number ‘SYXK2002-006’ for this study), Beijing, China.

### Thermal injury with *P. aeruginosa* infection model

A mouse full-thickness skin burn model was reproduced. Briefly, mice were subjected to a 10% scald injury on the abdominal surface, and were subjected to intraperitoneal inoculation of *P. aeruginosa* (2×10^5^ CFU/ml) on postburn day(PBD) 1. Then, mice were given intravenous injection of APS(50, 100, and 200 mg/kg) on PBD 2, 4 and 6, respectively. Mice that received scald injury only and healthy mice served as controls.

### Experimental design

One hundred sixty-eight mice were randomly divided into six groups as follows: sham burn group (28 mice), burn control (burn without infection animals) group (28 mice), burn plus *P. aeruginosa* group (28 mice), burn plus *P. aeruginosa* with APS(50 mg/kg) treatment group (28 mice), burn plus *P. aeruginosa* with APS(100 mg/kg) treatment group (28 mice), and burn plus *P. aeruginosa* with APS(200 mg/kg) treatment group (28 mice). All of these groups were further divided into four subgroups of seven mice each, and they were sacrificed on PBD 1, 3, 5 and 7, respectively. In addition, 7 mice were taken to serve as normal controls (the main parameters determined in the current study were found to be highly constant in 7 mice of sham burn animals on PBD 0, thus the results were shown as sham burned mice) sacrificed on PBD 0. Animals of all groups were sacrificed at designated time points, and peripheral blood samples were collected to procure Tregs and T cells immediately.

Another 75 mice of burn group, burn plus *P. aeruginosa* group, and APS treatment groups were employed to evaluate the effect of APS on the mortality rate following thermal injury combined with *P. aeruginosa* infection: burn group (15 mice), burn plus *P. aeruginosa* group (15 mice), burn plus *P. aeruginosa* with APS (50 mg/kg) treatment group (15 mice), burn plus *P. aeruginosa* with APS (100 mg/kg) treatment group (15 mice), and burn plus *P. aeruginosa* with APS (200 mg/kg) treatment group (15 mice), respectively.

### Reagents and kits

RPMI 1640, fetal calf serum (FCS), glutamine, penicillin, streptomycin, and HEPES were purchased from TianRunShanda Biotech Co. Ltd, Beijing, China. Mouse CD4^+^CD25^+^Treg MicroBeads were purchased from Miltenyi Biotec GmbH, Bergisch Gladbach, Germany. Thiazolyl blue (MTT) and TritonX-100 were purchased from Sigma, St. Louis, MO. Antibodies used for flow cytometric analysis, including fluorescein isothiocyanate (FITC)-conjugated anti-mouse CD4 and TLR4, phycoerythrin (PE)-conjugated anti-mouse CD25 and allophycocyanin (APC)-conjugated anti-mouse Foxp3, were purchased from BD/PharMingen, San Diego, CA. Functional purified grade anti-mouse CD3 and CD28 were purchased from BD/PharMingen, San Diego, CA. Total RNA isolation system and reverse transcription (RT) system were purchased from Promega, Madison, WI. SYBR Green PCR Master MIX was purchased from Applied Biosystems, Foster City, CA. Enzyme-linked immunosorbent assay (ELISA) kits of murine IL-10, IL-2, IL-4, and interferon (IFN)-γ were purchased from Biosource, Worcester, MA.

### Isolation of CD4^+^CD25^+^Tregs from peripheral blood

Peripheral blood was obtained in 3–5 ml of RPMI 1640 and treated with erythrocytolysin. After centrifugation, the sedimentary cells were collected. The cells were isolated using anti-Treg (CD4/CD25) MicroBeads and a MiniMACS™ separator according to manufacturer's instructions. CD4^+^ T cells were enriched by depletion of cells expressing CD8α, CD11b, CD45R, CD49b and Ter-119 from peripheral blood with a CD4^+^CD25^+^ Regulatory T Cell Isolation Kit (Miltenyi Biotec GmbH, Bergisch Gladbach, Germany). CD4^+^CD25^+^Tregs and CD4^+^CD25^−^T cells were further selected according to the expression of CD25. The purity of isolated CD4^+^CD25^+^Tregs was verified by flow cytometric analysis with FITC-conjugated anti-CD4 and PE-conjugated anti-CD25 staining.

### Purification of CD4^+^ T cells from peripheral blood

The CD4^+^ T Cell Isolation Kit (Miltenyi Biotec GmbH, Germany) was an indirect magnetic labeling system for the isolation of untouched CD4^+^ T cells from suspensions of murine peripheral blood cells. Non-CD4^+^ T cells, i.e. cytotoxic T cells, B cells, natural killer (NK) cells, dendritic cells (DCs), macrophages, granulocytes and erythroid cells were indirectly magnetically labeled by using a cocktail of biotin-conjugated antibodies against CD8a (Ly-2), CD45R (B220), DX5, CD11b (Mac-1) and Ter-119, and anti-biotin MicroBeads. Isolation of highly pure CD4^+^ T cells was achieved by depletion of magnetically labeled cells.

### Cell culture and cytokine measurements

Supplemented RPMI 1640 consisted of RPMI 1640 with 10% FCS. All cells were cultured at 37°C in 5% CO_2_. CD4^+^CD25^+^ Tregs as well as CD4^+^ T cells isolated from the peripheral blood of male BABL/c mice by magnetic beads were seeded on 48-well (1×10^5^ CD4^+^CD25^+^ Tregs/well and 1×10^6^ CD4^+^ T cells/well) cell culture plates coated with anti-CD3 (1 µg/ml) and soluble anti-CD28 (1 µg/ml). The dose-dependent responses between APS stimulation and TLR4 expression on CD4^+^CD25^+^ Tregs were analyzed by flow cytometry. After being stimulated with APS for 24 h or in the dose of 200 µg/ml, secretion of IL-10 in Tregs as well as Foxp3 mRNA of Tregs were determined. The supernatants were collected from each well for determination of IL-2, IL-4, and IFN-γ levels with ELISA kits, strictly following the protocols provided by the manufacturers. The color reaction was terminated by adding 100 µl of ortho-phosphoric acid. Plates were read in a microplate reader (Spectra MR, Dynex, Chantilly, VA). All samples were run in guadruplicates.

### CD4^+^CD25^+^ Tregs proliferation/suppression assay

CD4^+^ T cells were suspended in RPMI 1640 culture medium supplemented with 10% FCS, 100 U/ml penicillin, 100 µg/ml streptomycin, and placed in 48-well round bottom plates for proliferation, giving the final cell density of 2×10^6^/ml with CD4^+^CD25^+^ Tregs in ratio of 1∶10 (Tregs to CD4^+^ T cells). Cells were treated with 1 µg/ml anti-CD3 for 68 h at 37°C in 5% CO_2_/100% humidified air. 100 µl supernatant was decanted from each well while 10 µl thiazolyl blue (MTT, 5 mg/ml) was added. After cultured for another 4 h, 100 µl Triton-Isop (10% TritonX-100, 50% Isopropanol, 0.01 mol/L HCl) was added into each well, and the proliferation of CD4^+^ T cells was measured by detection of optical density with a microplate reader (Spectra MR, Dynex, Chantilly, VA) at the wavelength of 540 nm (OD540 nm). All samples were run in quadruplicates. After being stimulated for 72 h with 200 µg/ml APS. Proliferative activity of CD4^+^ T cells was analyzed by above mentioned MTT test. To examine the effects of CD4^+^CD25^+^ Tregs on responder cells, cytokines of Th responses were measured. After CD4^+^CD25^+^ Tregs and CD4^+^ T cells were cultured in cell-ratio of 1∶10 for 72 h, the supernatants were removed for measurement of various cytokines, including IL-4 and IFN-γ by means of ELISA. All samples were run in quadruplicates.

### SYBR Green real-time RT-PCR

Approximately 2×10^5^ cells/sample were prepared for extracting total RNA using the single-step technique of acid guanidinium thiocyanate-chloroform extraction according to the manufacturer's instruction. The concentration of purified total RNA was determined spectrophotometrically at 260 nm. mRNA expressions for Foxp3 and glyceraldehyde-3-phosphate dehydrogenase (GAPDH) in cells were quantified by SYBR Green two-step, real-time RT-PCR. After removal of potentially contaminating DNA with DNase I, 1 µg of total RNA from each sample was used for reverse transcription with an oligo dT and a Superscript II to generate first-strand cDNA. PCR reaction mixture was prepared using SYBR Green PCR Master Mix. The primer sequences were as follows: GAPDH, forward: 5′-TTCACCACCATGGAGAAGGC -3′, and reverse: 5′-GGCATGGACTGTGGTCATGA-3′; Foxp3, forward: 5′-CAGCTGCCTACAGTGCCCCTAG-3′, and reverse: 5′-CATTTGCCAGCAGTGGGTAG-3′. The amplification PCR consisted of 1 min denaturation step at 95°C followed by 40 cycles of 15 sec at 95°C and 40 sec at 60°Con a Sequence Detection System (Applied Biosystems, Foster City, CA). All samples were run in quadruplicates.

### Flow cytometry

To observe the CD4 and CD25 expression on the surface of Tregs, cells were stained with hamster anti-mouse CD4-FITC antibody and CD25-PE antibody for 30 min at 4°C in darkness. Concomitantly, for detection of intranuclear Foxp3, cells were reacted with 1 ml freshly prepared fixation/permeabilization working solution for 2 h at 4°C. After washing cells with permeabilization buffer twice, cells were stained with anti-mouse/rat Foxp3-APC antibody (eBioscience, San Diego, CA) for 30 min at 4°C in the dark. After washing twice, cells were analyzed by flow cytometry using a FACScan (BD Biosciences, Mountain View, CA).

### Statistical analysis

Data were expressed as mean ± standard deviation (SD). Data were analyzed with software of SPSS14.0 and tested by analysis of a one-way variance (ANOVA) test and logrank test. P values below 0.05 or 0.01 were considered significant.

## Results

### Effects of APS on phenotypic changes in CD4^+^CD25^+^ Tregs *in vivo*

**Figure 1:**
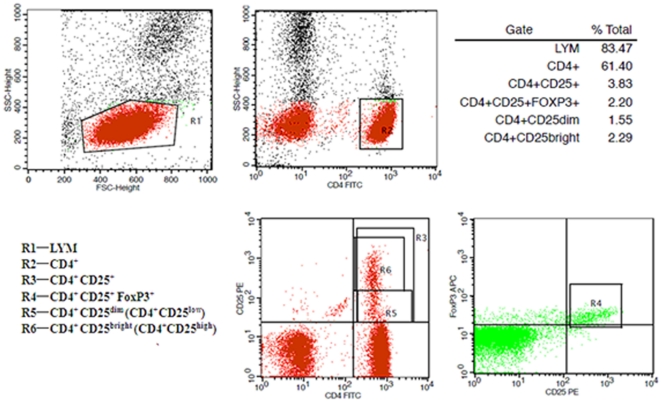
Phenotypic expressions on Tregs. Peripheral lymphocytes were analyzed for CD4-FITC, CD25-PE and Foxp3-APC expressions. The graphs showed that CD4^+^ T cells gated by R2 were isolated from lymphocytes and divided into CD4^+^CD25^+^(R3), CD4^+^CD25^+^Foxp3^+^ (R4), CD4^+^CD25^low^ (R5) and CD4^+^CD25^high^ (R6). Data represented the results of one representative experiment out of seven performed under the same conditions.

**Figure 2:**
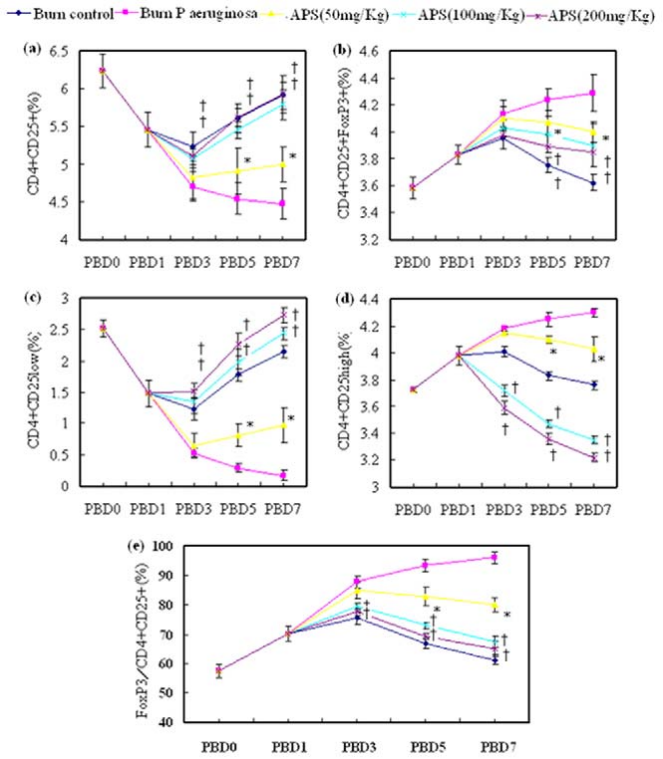
Changes of phenotype in CD4^+^CD25^+^ Tregs induced by APS. The ratios of CD4^+^CD25^+^/CD4^+^, CD4^+^CD25^+^Foxp3^+^/CD4^+^, CD4^+^CD25^low^/CD4^+^, CD4^+^CD25^high^/CD4^+^ and Foxp3^+^/CD4^+^CD25^+^ of sham burn mice, burn control mice, burn plus *P. aeruginosa* and burn plus *P. aeruginosa* with APS (50,100, and 200 mg/kg) treatment on PBD 1, 3, 5 and 7 were detected with FACS analysis. * *P*<0.05 compared with the burn plus *P. aeruginos* group by the one-way ANOVA test. †*P<*0.05 compared with mice received APS (50 mg/kg) by the one-way ANOVA test.

To investigate the effects of APS on peripheral blood Tregs in terms of dynamics in phenotypic expression of Foxp3 as well as CD4^+^CD25^+^, CD4^+^CD25^low^ and CD4^+^CD25^high^ as shown in Fig. 1, these cells were analyzed at different time points in animals with sham burn, burn, burn plus *P. aeruginosa*, or burn plus *P. aeruginosa* with APS (50,100 and 200 mg/kg) treatment. As shown in Fig. 2, the expression levels of Foxp3 and CD4^+^CD25^high^ were slowly enhanced on Tregs in the burn group during PBD 1 to 7 compared with the sham injured group, while the expression of Foxp3 and CD4^+^CD25^high^ in the burn plus *P. aeruginosa* group were markedly increased on Tregs during PBD 3 to 7 compared with the sham burn group (P<0.01). Treatment with APS could significantly decrease the expression levels of Foxp3 and CD4^+^CD25^high^ on Tregs (P<0.05), and the down-regulatory effect of APS was observed to manifest in a dose-dependent fashion.

Surprisingly, as shown in Fig. 2, the expression levels of CD4^+^CD25^+^ and CD4^+^CD25^low^ in the burn group were gradually decreased on Tregs during PBD 1 to 7 compared with the sham burn group, while the expression of CD4^+^CD25^+^ and CD4^+^CD25^low^ on Tregs in the burn plus *P. aeruginosa* group were notably weakened during PBD 3 to 7 compared with the sham burn group (P<0.01). Treatment with APS could significantly enhance the expression levels of CD4^+^CD25^+^ and CD4^+^CD25^low^ on Tregs (P<0.05), and the up-regulatory effect of APS was observed in a dose-dependent pattern.

### Burn or burn with *P. aeruginosa* infection induced T cell activation and polarization *in vivo*

To understand the mechanism concerning the involvement of Tregs in the effect of APS on CD4^+^ T cell-mediated immunity, CD4^+^ T-cell proliferative activity and production of cytokines were analyzed *ex vivo*. The CD4^+^ T-cell proliferative activities in response to CD3 and CD28 in the burn group were moderately suppressed during PBD 1 to 7 as compared with the sham controls. In the burn plus *P. aeruginosa* group, however, the CD4^+^ T-cell proliferative activities in response to CD3 and CD28 were markedly suppressed during PBD 3 to 7 as compared with the sham burn group. The suppression of burn plus *P. aeruginosa* infection could be reverted by treatment with APS (P<0.01) in a dose-dependent fashion. Secretion of IL-2 by T cells were simultaneously suppressed to a certain extent after burn injury with *P. aeruginosa* infection (P<0.01), and APS treatment could notably restore IL-2 release by T cells (P<0.01; Fig. 2).

**Figure 3:**
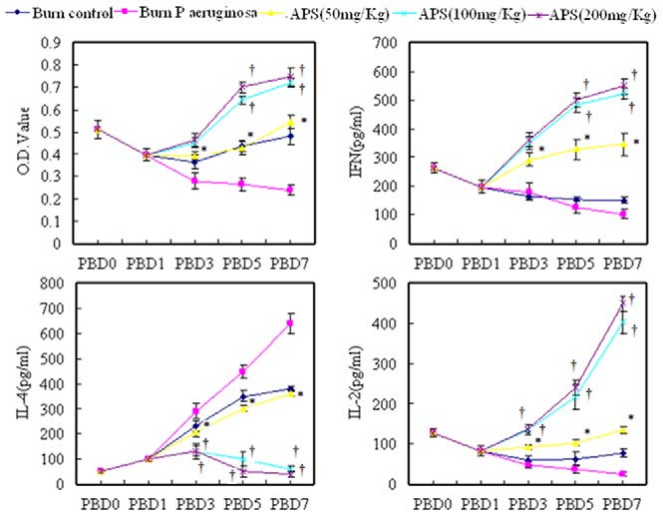
T cell activation and polarization after injury *in vivo*. The IL-2, IL-4 and IFN-γ secretion of CD4^+^ cells and O.D. value of CD4^+^ cells separated from sham burn mice, burn control mice, burn plus *P. aeruginosa* and burn plus *P. aeruginosa* with APS (50, 100, and 200 mg/kg) treatment on PBD 1, 3, 5, and 7 were detected by MTT or ELISA in *ex vivo* culture. * *P*<0.05 compared with the burn plus *P. aeruginosa* group by the one-way ANOVA test. †*P<*0.05 compared with mice received APS (50 mg/kg) by the one-way ANOVA test.

It is well known that Th1 cells produce IFN-γ, and Th2 cells produce IL-4. Thus, we detected the these cytokines produced by CD4^+^ T cells to identify polarization of Th1/Th2. After burn injury with *P. aeruginosa* infection, the levels of IL-4 produced by CD4^+^ T cells in response to CD3 and CD28 were markedly increased (P<0.01), peaking on PBD 7 (P<0.01; Fig. 3), whereas the levels of IFN-γ significantly lowered (P<0.01) in comparison to burned mice as well as sham burn group, indicating that CD4^+^ T cells had developed into Th2 cells. Treatment with APS could significantly inhibit elevation of levels of IL-4 (P<0.01) and reduced the levels of IFN-γ (P<0.01) after thermal injury with *P. aeruginosa* infection, indicating that treatment with APS might influence the polarization of CD4^+^ T cells in burned mice with *P. aeruginosa* infection and induced naive CD4^+^ T cells to shift to Th1 cells.

### APS protected burned mice with *P. aeruginos* infection against lethality

**Figure 4:**
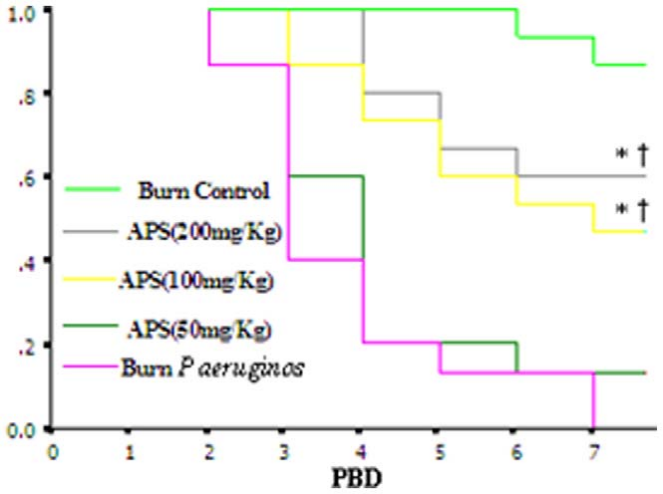
Protective effect of APS against lethality induced by burned mice with *P. aeruginos* infection. BALB/c mice (15/group) were subjected to 10% TBSA scald injury, and intraperitoneal inoculation of *P. aeruginosa* (2×10^5^ CFU/ml) on PBD 1. Then, mice received intravenous injection of APS (50, 100, or 200 mg/kg) on PBD 2, 4 and 6. *P<0.01 compared with the burn plus *P. aeruginos* group by the log-rank test. †*P<*0.01 compared with mice received APS (50 mg/kg) by the log-rank test.

To clarify the protective response of APS against lethality induced by *P. aeruginosa* challenge, we investigated the effect of APS on reduction of mortality rate of thermal injury with intraperitoneal inoculation of *P. aeruginos* on PBD 1. Intravenous injection of APS (50 mg/kg) on PBD 2, 4, and 6 after intraperitoneal inoculation of *P. aeruginos* to mice with 10% TBSA scald injury slightly reduced the mortality rate (Fig. 4). However, treatment with APS (100 and 200 mg/kg) on PBD 2, 4 and 6 after *P. aeruginos* challenge to 10% TBSA scald mice significantly decreased the mortality rates, and the preventive effect was observed to manifest in a dose-dependent fashion (Fig. 4).

### APS inhibited the role of CD4^+^CD25^+^Tregs in negative immune regulation via down-regulation of TLR4 *ex vivo*

TLRs have been shown to be involved in the antibacterial defense in humans. To determine the involvement of these receptors in the interaction of CD4^+^CD25^+^Tregs with APS, neutralization experiments were performed in the present study. Cell-surface TLR-4 receptor was blocked by neutralizing concentrations of its respective antibody before CD4^+^CD25^+^Tregs were treated with APS. We showed positive and negative controls for the neutralization of TLR-4 (by using anti-TLR4 and TLR4-isotype).

**Figure 5:**
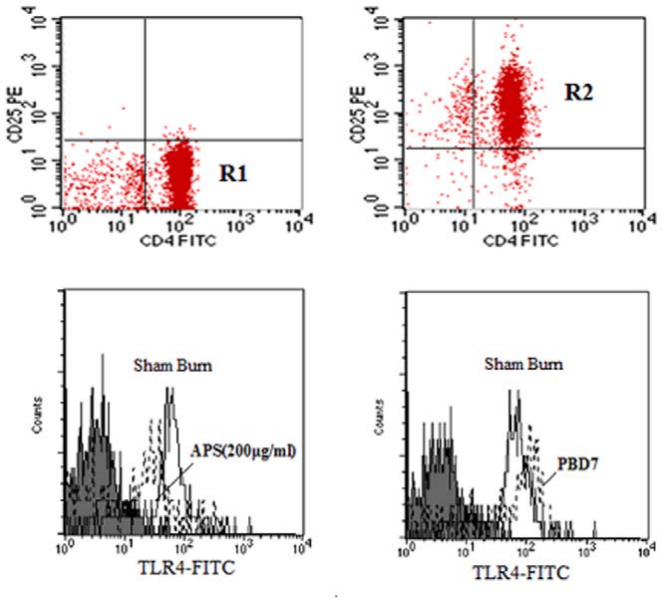
Changes of TLR4 expression on CD4^+^CD25^+^Tregs. CD4^+^CD25^+^Tregs (R2) and CD4^+^CD25^−^T cells (R1) were isolated from the peripheral blood of male BALB/c mice by magnetic beads and analyzed by flow cytometry. The expression of TLR4 on CD4^+^CD25^+^Tregs was stained with the indicated FITC-antibodies. Data were represented by a histogram in which gray area represents isotype-matched control mAbs, solid lines represent CD4^+^CD25^+^Tregs isolated from sham burn mice, and dotted lines represent CD4^+^CD25^+^Tregs also stimulated with APS(200 µg/ml) for 24 hr or isolated from burn plus infection mice at PBD7. Data represent the results of one representative experiment out of seven.

**Figure 6:**
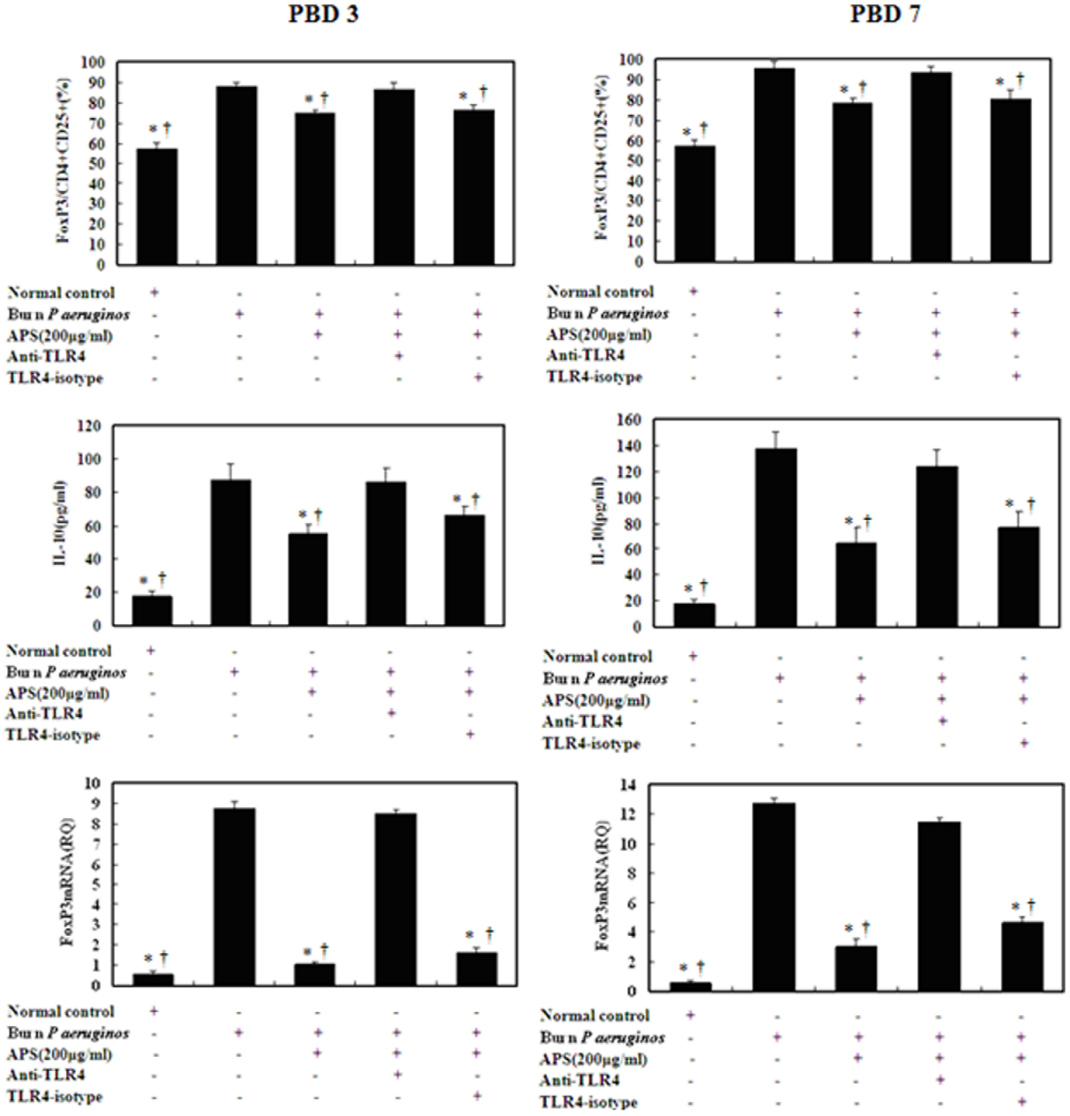
The inhibition of APS on negative immune regulation of CD4^+^CD25^+^Tregs via TLR4 down-regulation *ex vivo*. CD4^+^CD25^+^Tregs were isolated from sham burn mice as normal controls and burn plus *P. aeruginos*, and subsequently, induced by APS (200 µg/ml). Compared to the reversal of APS by anti-TLR4 mAb, the administration of isotype control antibody could not inhibit the effect of APS on CD4^+^CD25^+^Tregs. Data were expressed as mean ± SD of samples. *P<0.01, compared to CD4^+^CD25^+^Tregs isolated from burned mice with *P. aeruginos* infection; † P<0.01, compared to CD4^+^CD25^+^Tregs isolated from burn plus *P. aeruginos* group treated by APS with anti-TLR4 antibody.

As shown in Fig. 5, the purity of CD4^+^CD25^+^Tregs and CD4^+^CD25^−^T cells were 92%∼98% and 90%∼95% respectively. After stimulation with APS in the dosages of 200 µg/ml, TLR4 expression on CD4^+^CD25^+^Tregs were markedly down-regulated at 24 h. Without APS stimulation, the TLR4 expression on CD4^+^CD25^+^Tregs isolated from burn plus infection mice were significantly increased on PBD7. (Fig. 5). When treated with APS (200 µg/ml) for 24 h *ex vivo*, the intranuclear Foxp3 protein, Foxp3 mRNA expressions and IL-10 secretion of CD4^+^CD25^+^Tregs isolated from burn plus infection mice on PBD 3 and 7 were obviously lower than those in untreated burn plus infection mice on PBD 3 and 7 (P<0.05). (Fig. 6). To further determine whether TLR4 played a potential role consequent to the inhibitory effect of APS on CD4^+^CD25^+^Tregs, we investigated changes in intranuclear Foxp3 protein, Foxp3 mRNA expressions, and IL-10 secretion of APS-Tregs with anti-TLR4 and isotype of TLR4 on PBD 3 and 7. It was found that blockade of TLR4 markedly diminished the suppressive capacity of APS on CD4^+^CD25^+^Tregs in terms of expression as well as secretion of Foxp3 and IL-10 on PBD 3 and 7.

### APS activated CD4^+^ T cells and directed Th1 polarization via TLR4 on CD4^+^CD25^+^Tregs *in vitro*

**Figure 7:**
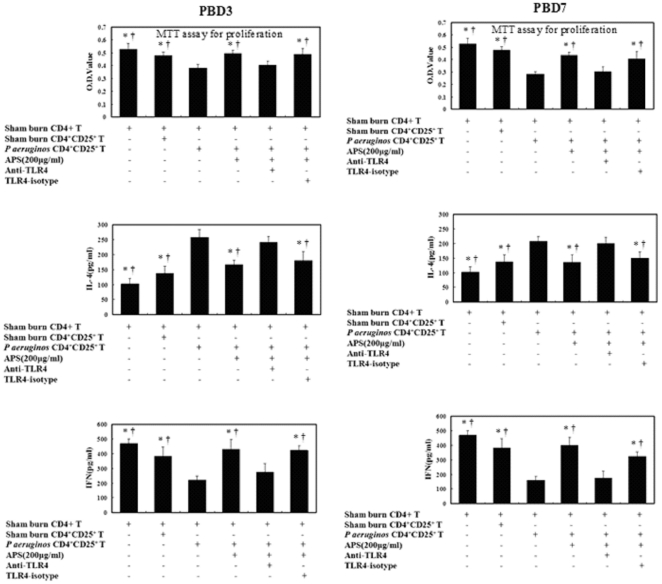
The effect of APS on activation and polarization of CD4^+^ T cells via TLR4 on CD4^+^CD25^+^Tregs *in vitro*. Compared to the reversal of APS by anti-TLR4 mAb, the administration of isotype control antibody could not inhibit the effect of APS on activation and polarization of CD4^+^ T cells in mixed lymphocyte reaction of CD4^+^CD25^+^Tregs and CD4^+^ T cells. Data were expressed as mean ± SD of samples. * P<0.01, compared to CD4^+^CD25^+^Tregs isolated from burned mice with *P. aeruginos* infection; † P<0.01, compared to CD4^+^CD25^+^ Tregs isolated from burn plus *P. aeruginos* group treated by APS with anti-TLR4 antibody.

To investigate if TLR4 on CD4^+^CD25^+^Tregs mediated the effect of APS on activation and polarization of CD4^+^T cells, we performed mixed lymphocyte reaction (MLR) in terms of CD4^+^CD25^+^ Tregs and CD4^+^T cells *ex vivo*. After APS stimulation, CD4^+^CD25^+^Tregs were separated from APS+CD4^+^CD25^+^Tregs, APS+anti-TLR4+CD4^+^CD25^+^Tregs and APS+TLR4-isotype+CD4^+^CD25^+^Tregs mixed culture, and subsequently, used to administrate CD4^+^CD25^+^ Tregs + CD4^+^ T cells MLR. Compared with the inhibition of CD4^+^ T cell proliferation with shifting from Th2 to Th1 in MLR of APS + CD4^+^CD25^+^Tregs + CD4^+^T cells (CD4^+^CD25^+^Tregs: CD4^+^T = 1∶100), anti-TLR4 + APS could markedly suppress CD4^+^ T cell proliferation and reverse CD4^+^ T cell differentiation in mixed lymphocyte reaction. To further clarify whether TLR4 contributed to the regulatory activity of APS, we used anti-TLR4 antibody as well as isotype mAbs in the the current study. It was revealed that blockade of TLR4 notably reduced the stimulatory capacity of APS as shown in Fig. 6 and Fig. 7.

## Discussion

In recent studies, APS has been shown to potentiate the immune activity of IL-2 and the activity of monocytes, improve the responses of lymphocytes obtained from normal subjects and immunosuppressed patients. To investigate the immune regulation of the flavonoids of *Astragalus membranaceus*, mAb assays of changes in total T-cell count and subsets were performed before and after treatment in immunosuppressed mice. Results indicated that the flavonoids could promote the proliferation of lymphocytes, raise the T-cell count, regulate the T-cell subsets. Likely, experiments in human cell culture have been demonstrated that APS is also active on human lymphocytes. In addition, it was reported that APS was mitogenic to T cell-depleted lymphocyte population but virtually inactive on B cell-depleted population [24]. On the basis of these findings, the investigators clearly stated that the immunomodulatory effects of APS were extensive, including promoting both humoral and cellular immunity, improving the immune functions in a murine immunosuppressive model, and modulating the production of cytokines [25]. More recently, in an *in vitro* study we found that APS could induce the differentiation of splenic DCs to IL-12-producing CD11c^high^CD45RB^low^DCs, and further mediate the activation of immune function of CD4^+^ T cells with shifting of Th2 to Th1 [26]. However, it is not clear whether APS can modulate the function of naturally occurring peripheral blood CD4^+^CD25^+^Tregs in mice followed by activating T cell-mediated immunity. Herein, we showed that APS could inhibit the function of circulating CD4^+^CD25^+^Tregs, thereby mediating activation of T lymphocyte immune function and shifting of Th2 to Th1 via down-regulating TLR4 expression on CD4^+^CD25^+^Tregs, which were isolated from burned mice with *P. aeruginosa* infection *in vivo* and *ex vivo*.

The immune response to sepsis presents a complex balance between the successful induction of proinflammatory antipathogen responses and anti-inflammatory responses required to limit damage to host tissues. Tregs undoubtedly play important roles in controlling this balance during serious infection or sepsis, and the results can range from highly detrimental to the host to highly beneficial to both the host and pathogen. In our current observations, significant suppression of T cell proliferation during PBD 1 to 7 was found, and expression levels of IL-2 in T-cell supernatant and IL-2Rα (CD25) on the CD25^low^/CD25^−^ T-cell surface were simultaneously suppressed to a certain extent. IL-2Rα expressed on the CD25^high^ T-cell surface, however, was remarkably enhanced during PBD 1 to 7 with simultaneous elevation in Foxp3 expression in burned mice with *P. aeruginosa* challenge. It was also revealed that T cells were polarized to Th2 cells after burn with *P. aeruginosa* infection. The previously discussed data indicated that there was a marked suppression of T cells after major burns followed by sepsis [27]. Our results collaborate with reports of other authors that Tregs in mice can inhibit the proliferation of T cells and release of cytokine for polarization to antigen-specific Th1 cells after burn with *P. aeruginosa* challenge [28]. A classic characteristic of Tregs is their lack of proliferative response upon T cell receptor activation or stimulation with mitogenic antibodies. Thus, CD4^+^CD25^high^Tregs depend on exogenous IL-2 but inhibit the transcription of IL-2 as well as IL-2Rα in conventional CD25^low^/CD25^−^ T cells [29].

In the present study, it was demonstrated that APS could down-regulate the suppressive activity of CD4^+^CD25^+^Tregs, which was associated with marked decrease in Foxp3 expression of peripheral blood Tregs both *in vivo* and *ex vivo*. Concomitantly, Foxp3 and IL-10 mRNA expressions were noticed showing a similar pattern as protein expression of Foxp3. It has been reported that the function of Tregs is governed by Foxp3, and lacking this expression, Treg function is lost [30], whereas transduction of Foxp3, which is special transcription factors in the development and function of natural Tregs, induces the expression of CD25^high^, cytotoxic T lymphocyte-associated antigen 4 [31], and it exerts anergic and suppressive activity *in vitro*. IL-10 is expressed by various immune cells, including T cells, B cells, and macrophages. Its wide range of immunosuppressive effect includes lowering of T-cell cytokine production, inhibition of antigen presentation, down-regulation of expression of costimulatory molecules on antigen presenting cells, and direction of T-cell differentiation into Th2 [32]. In this study, the cytokine secretion of these CD4^+^CD25^+^Tregs was detected, and it was revealed that CD4^+^CD25^+^Tregs produced higher levels of IL-10 after burn injury combined with *P. aeruginosa* infection. A similar phenomenon was also noted in cecal ligation and puncture-induced sepsis in rats [27] and patients with septic shock [33].

In the APS-stimulated group, IFN-γ level was elevated in the supernatant of CD4^+^ T cells, while IL-4 level was lowered in the supernatant of CD4^+^ T cells isolated from mice peripheral blood in comparison with unstimulated group. In the *ex vivo* experiments, proliferative activity of CD4^+^ T cells was markedly suppressed when the ratio of CD4^+^CD25^+^Tregs to CD4^+^ T cells was 1∶10, meanwhile, the proliferation of CD4^+^ T cells was enhanced when cultured with APS-stimulated CD4^+^CD25^+^Tregs. These data suggested that APS appears to be involved in modulating T cell-mediated immunity by influencing proliferation of effector T cells and cell-polarization. It is well known that the T helper cells are divided into two subsets when activated, i.e., Th1 cells secrete IFN-γ while the Th2 cells secrete IL-4 [34]. CD4^+^CD25^+^Tregs isolated from burned mice with *P. aeruginosa* infection induced the response of evoking Thl shifting to Th2 as shown in our study, and, when CD4^+^ T cells were cultured with APS-stimulated CD4^+^CD25^+^Tregs, Th2 subset could shift to Th1 subset. Although a previous report suggested that APS could induce the differentiation of DCs to IL-12-producing CD11c^high^CD45RB^low^DCs, and further induce activation of immune function of T lymphocyte with shifting of Th2 to Th1 *in vitro*, our current expereiment implied that CD4^+^CD25^+^Tregs might react with effector T cells by a direct interaction. Nevertheless, there are several mechanisms concerning Tregs-mediated suppression both *in vivo* [35] and *in vitro*, including down-regulation of the CD80 and major histocompatibility complex-II expression on DCs, secretion of suppressive cytokines, and induction of apoptosis of T cells [36]. The present study provided new insight into the important role of APS in the modulation of CD4^+^CD25^+^Tregs response to corroborate the above findings.

To further clarify the potential receptor mechanism of APS binding to mature CD4^+^CD25^+^Tregs *ex vivo*, we used anti-TLR4 antibody to block TLR4 in this experiment. It was found that there was a markedly up-regulated expression of TLR4 on the surface of CD4^+^CD25^+^Tregs from burned mice with *P. aeruginosa* infection. TLR4 was blocked by treatment with anti-TLR4 antibody, resulting in a significant decrease of expression of Foxp3 on CD4^+^CD25^+^Tregs together with lowered levels of IL-10 production. These results suggested that the effect of APS on CD4^+^CD25^+^Tregs was inhibited by blockade of TLR4, and the influence of APS in inducing maturation of CD4^+^CD25^+^Tregs could be mediated through manipulation of TLR4 signaling, thus linking this pathway to the control of Tregs function. In addition, we noted that administration of anti-TLR4 antibody remarkably restored T-cell proliferative activity and expression levels of IL-2 as well as IL-2Rα after burn injury with *P. aeruginosa* infection, and could significantly initiate T-cell shift to Th1 cells.

In conclusion, APS might suppress CD4^+^CD25^+^Treg activity, at least in part, via binding TLR4 on the surface of CD4^+^CD25^+^Tregs, and trigger a shift of Th2 to Th1 with activation of CD4^+^ T cell-mediated immunity in burned mice combined with *P. aeruginosa* infection. Further investigation is warranted to define the signaling transduction of APS on CD4^+^CD25^+^Treg activity in postburn sepsis.
