# Correction: Astragalus Polysaccharides Attenuate Postburn Sepsis via Inhibiting Negative Immunoregulation of CD4^+^CD25^high^ T Cells

**DOI:** 10.1371/annotation/6c65352a-a393-4130-98b4-9a39793723d6

**Published:** 2011-07-05

**Authors:** Qing-yang Liu, Yong-ming Yao, Yan Yu, Ning Dong, Zhi-yong Sheng

A grant was assigned incorrectly to the third author. The grant was issued to the second author, Yong-ming Yao. The correct funding statement is: "This study was supported, in part, by grants from the National Natural Science Foundation of China (http://www.nsfc.gov.cn/Portal0/default124.htm)(grant no. 81000847 to Q-yL; 81071545 to Yong Ming Yao), and the National Basic Research Program of China (http://www.973.gov.cn/)(grant no. 2005CB522602 to Yong Ming Yao). The funders had no role in study design, data collection and analysis, decision to publish, or preparation of the manuscript."

